# Trichosanthin Inhibits Breast Cancer Cell Proliferation in Both Cell Lines and Nude Mice by Promotion of Apoptosis

**DOI:** 10.1371/journal.pone.0041592

**Published:** 2012-09-05

**Authors:** Evandro Fei Fang, Chris Zhi Yi Zhang, Lin Zhang, Jack Ho Wong, Yau Sang Chan, Wen Liang Pan, Xiu Li Dan, Cui Ming Yin, Chi Hin Cho, Tzi Bun Ng

**Affiliations:** 1 School of Biomedical Sciences, Faculty of Medicine, The Chinese University of Hong Kong, Shatin, Hong Kong; 2 State Key Laboratory of Oncology in Southern China, Sun Yat-Sen University Cancer Center, Guangzhou, China; 3 Department of Pathology, Sun Yat-Sen University Cancer Center, Guangzhou, China; University of Kansas Medical Center, United States of America

## Abstract

Breast cancer ranks as a common and severe neoplasia in women with increasing incidence as well as high risk of metastasis and relapse. Translational and laboratory-based clinical investigations of new/novel drugs are in progress. Medicinal plants are rich sources of biologically active natural products for drug development. The 27-kDa trichosanthin (TCS) is a ribosome inactivating protein purified from tubers of the Chinese herbal plant *Trichosanthes kirilowii* Maximowicz (common name Tian Hua Fen). In this study, we extended the potential medicinal applications of TCS from HIV, ferticide, hydatidiform moles, invasive moles, to breast cancer. We found that TCS manifested anti-proliferative and apoptosis-inducing activities in both estrogen-dependent human MCF-7 cells and estrogen-independent MDA-MB-231 cells. Flow cytometric analysis disclosed that TCS induced cell cycle arrest. Further studies revealed that TCS-induced tumor cell apoptosis was attributed to activation of both caspase-8 and caspase-9 regulated pathways. The subsequent events including caspase-3 activation, and increased PARP cleavage. With regard to cell morphology, stereotypical apoptotic features were observed. Moreover, in comparison with control, TCS- treated nude mice bearing MDA-MB-231 xenograft tumors exhibited significantly reduced tumor volume and tumor weight, due to the potent effect of TCS on tumor cell apoptosis as determined by the increase of caspase-3 activation, PARP cleavage, and DNA fragmentation using immunohistochemistry. Considering the clinical efficacy and relative safety of TCS on other human diseases, this work opens up new therapeutic avenues for patients with estrogen-dependent and/or estrogen-independent breast cancers.

## Introduction

Among 145 families of plants, the plant genus *Trichosanthes* (family Cucurbitaceae) comprises vines which own good potential as the target for anticancer drug discovery [1]. Within this genus, *Trichosanthes kirilowii* Maximowicz is the most well-known medicinal species for there is a long history for the clinical application as well as experimental studies of its tubers [2]. In the Chinese ethnomedicine *T. kirilowii* tuber, well-known by its vernacular name ‘Tian Hua Fen’, is considered as a ‘wonder drug’ against some gynecological conditions/illnesses. It was used as an abortifacient and for the treatment of hydatidiform moles, invasive moles, and ectopic pregnancy [2], [3], [4]. These effects have been confirmed by empirical studies for the mainly medicinal effects are attributed to trichosanthin (hereafter referred to as TCS), a 27-kDa protein purified from *T. kirilowii* tubers. The mature form of TCS contains 247 amino acids, but its preprotein consists of 289 amino acids with a 23-residue N-terminal signal peptide and a 19-residue C-terminal propeptide [2], [5]. TCS shares remarkable sequence similarity with other antitumor proteins in plant genera *Trichosanthes* and *Momordica*, including trichomislin (27.2 kDa) and karasurin-C (27.4 kDa) from *T. kirilowii*, as well as MAP30 and the N-glycosidase part of *Momordica charantia* lectin/MCL purified from *M. charantia* [2], [6], [7].

Besides the ability to ablate the replication of human immunodeficiency virus (HIV) [8], [9], [10], [11] and herpes simplex virus type 1 (HSV-1) [12], TCS is a potential antidote against some tumors for it could compromise the tumor cell growth both *in vitro* and *in* vivo with different molecular mechanisms. First, TCS is useful for treating choriocarcinoma as it can produce reactive oxygen species (ROS) and initiate apoptosis in human choriocarcinoma (Jar) cells [13], [14], [15]. Second, TCS induced the death of human cervical carcinoma (Hela) cells by increasing cytosolic calcium, accompanied by the suppression of cAMP/protein kinase C levels [16], and by the damage of cytoskeleton configuration resulting from inhibited expression of actin and tubulin [17]. Third, the apoptosis-inducing activity of TCS applies to mouse NIH 3T3 embryonic fibroblasts by the activation of caspase-8 and caspase-3 involved pathways [18]. Fourth, TCS induced apoptosis in human lung cancer cells by G1 phase arrest, anti-telomerase effects, and inhibition of cell migration and metastasis [19], also previously observed in murine models [20]. Furthermore, TCS elicited caspase-3-mediated apoptosis in human leukemia HL-60 and K562 cells [21], [22]. Most recently, He and colleagues reported that TCS induced mitochondrial depolarization and caspase-9-based apoptosis in HSV-1 infected human laryngeal epidermoid carcinoma HEp-2 cells [23]. The specific tumor cell recognition as well as binding and cellular entry characteristics of TCS are based on its binging on different membrane proteins, such as a 2-macroglobulin receptor/LDL receptorrelated protein (LRP, 600 kDa) in trophoblasts, human choriocarcinoma Jar and human trophoblastic BeWo cells, and megalin in proximal tubule epithelial cells [24], [25], [26].

Breast cancer is one of the most prevalent and severe health problems for women [27]. Globally, the incidence was 0.641 million cases in 1980 which surged to 1.643 million cases in 2010, with an annual growth rate of 3.1% [28]. There were 425 000 women who died of breast cancer in 2010 [28]. It is heartening to observe that different new and promising components have been developed as the first-line therapy for breast cancer, but refractory obstacles such as metastasis and relapse make breast cancer still a big challenge ahead of us [29]. Here we report the anti-proliferative activity of TCS toward breast cancer cells in both *in vitro* and *in vivo* studies. The potential antitumor mechanisms were also unveiled. This work broadens the medicinal applications of TCS which may serve as a novel treatment against human breast cancer.

## Materials and Methods

### Purification of TCS

Dried pieces of the root tubers of *T. kirilowii* (origin: Guangdong Province in China) were purchased from a local vendor and authenticated by Prof. Shui-Ying Hu, Honorary Professor of Chinese Medicine at the Chinese University of Hong Kong (CUHK). TCS was purified according to the methods reported by Maraganore et al. [30]. Its purity was evaluated by 15% SDS-PAGE under reducing conditions [7].

### Cell culture

The two estrogen-dependent breast cancer cell lines used were MCF-7 cells, obtained from the American Type Culture Collection (ATCC) [31], and BT-474 cells obtained from cell services at Cancer Research UK (donated by Prof. Anthony Kong from University of Oxford) [32]. The MDA-MB-231 estrogen-independent breast cancer cell line (obtained from ATCC) was a kind gift of Prof. Kwok Pui Fung from the Chinese University of Hong Kong [33]. Human hepatocellular carcinoma Hep G2 cells were from ATCC and served as a positive control for TCS-induced cell death. All cells were grown in DMEM (Gibco) supplemented with 10% FBS and 1% penicillin/streptomycin, in an atmosphere of 5% CO2 at 37°C.

### Cell viability assay and cell proliferation assay

The effect of TCS on cell viability was performed by MTT assay as described before [7], [31]. Briefly, MCF-7, BT-474, and MDA-MB-231 cells were plated at a density of 5000 cells/well in 96-well plates and cultured overnight in DMEM medium. Serial two-fold dilutions of TCS at a final concentration ranging from 0.49∼125 µM were added to the respective wells. After 24 h or 48 h, the medium was discarded, followed by addition of 100 µL of 5 mg/mL MTT (Sigma) and incubation for another 2 h. Finally, 100 µL dimethyl sulfoxide was added to each well to dissolve the purple formazan. The absorbance was measured at 570 nm by using a BIO-RAD Microplate Reader. On the other hand, cell proliferation was analyzed by counting cell number with a haemocytometer using trypan blue exclusion as a criterion of cell viability.

### Assay of cell-cycle analysis

MCF-7 cells (TCS: 15 µM, 30 µM) and MDA-MB-231 cells (TCS: 10 µM and 20 µM) were treated with TCS at the abovementioned concentrations for 24 h. Subsequently, cells were harvested and fixed with ice-cold 70% ethanol for 2 h. Cells were then washed with PBS twice and stained with propidium iodide medium (50 µg/mL PI in PBS, containing 10 µg/mL RNase A) [7]. Finally, cells were loaded onto a FACSort flow cytometer (Becton Dickinson) with the data analyzed by FCS Express 4 software.

### Assessment of apoptosis, nuclear morphological changes, and DNA fragmentation

After culture with TCS for 24 h, MCF-7 cells (TCS: 15 µM, 30 µM) and MDA-MB-231 cells (TCS: 10 µM and 20 µM) were harvested and stained with 0.5 mg/ml Annexin V in binding buffer (10 mM HEPES free acid, 0.14 M NaCl, and 2.5 mM CaCl_2_) for 30 min. Afterward, PI (5 µg/mL final concentration) was added followed by incubation for another 15 min [7]. Data were recorded by using a FACSort flow cytometer (Becton Dickinson). On the other hand, TCS-induced cell morphological changes were studied by using the Hoechst 33342 staining dye (Sigma) and visualized under a NIKON TE2000 microscope [7]. Furthermore, DNA fragmentation was examined with a TUNEL staining based In Situ Cell Death Detection Kit (Roche) following the manufacturer's instructions [7]. Quantitative testing of activated caspase-8 and caspase-9 was performed by using caspase-8 colorimetric-kit and caspase-9 colorimetric-kit (Invitrogen, USA), respectively, according to the manufacturer's instructions.

### Assay of mitochondrial membrane depolarization

The loss of mitochondrial membrane potential, which is indicative of caspase 9-induced cell apoptosis, was measured by JC-1 staining [7]. After culture with TCS for 24 h, MCF-7 cells (TCS: 15 µM, 30 µM) and MDA-MB-231 cells (TCS: 10 µM and 20 µM) were harvested and stained with 2.5 µg/mL JC-1 dye (Sigma) for 15 min. Data were collected by using a FACSort flow cytometer (Becton Dickinson) and FCS Express 4 software was applied for data analysis.

### Western Blot

The effects of TCS on the regulation of caspase cascades were studied by Western blot as reported previously [7], [34]. After culture with TCS for 24 h, MCF-7 cells (TCS: 15 µM, 30 µM) and MDA-MB-231 cells (TCS: 10 µM and 20 µM) were harvested and lysed in ice-cold lysis buffer for 2 h, followed by centrifugation for 30 min at 4°C. Supernatants were aliquoted, heated and loaded on SDS-PAGE, following which proteins in gels were transferred to polyvinylidene difluoride membranes. Membranes were firstly blocked with 5% fresh milk for 1 h, followed by incubation with a corresponding primary antibody over night at 4°C. Finally, membranes were incubated with a horseradish peroxidase-conjudated anti-mouse or anti-rabbit secondary antibody (Cell Signaling, Danvers, MA) for 30 min, and bands were visualized by using ECL detection system (Amersham Life Science). The primary antibodies used in this study were as follows: antibodies fo tubulin (sc-9104, polyclonal), PARP (sc-25780, polyclonal) and cleaved PARP (sc-23461-R, polyclonal) were purchased from Santa Cruz Biotechnology (Santa Cruz, CA). Antibodies for caspase 8 (9746, monoclonal), caspase 9 (9508, monoclonal) and caspase 3 (9665, polyclonal) were from Cell Signaling (Danvers, MA).

### *In vivo* xenograft studies

Female BALB/c nude mice (seven weeks old, 20–25 g) were obtained from the Chinese University of Hong Kong Laboratory Animal Services Center, and procedures of all animal experiments had been approved by the Chinese University of Hong Kong Animal Research Ethics Committee. The estrogen-independent cell line MDA-MB-231 was employed for establishing the xenograft model following the procedures previously established for other tumor cells [7]. Firstly, 1×10^7^ MDA-MB-231 cells in 200 µL medium (100 µL DMEM medium +100 µL BD Matrigel™ Matrix) were inoculated subcutaneously into the right flanks of mice. Once the tumors were palpable, mice were randomly assigned into 2 groups (n = 6 per group, and with same tumor volume before TCS treatment). The TCS group received an intraperitoneal injection of sterile TCS in PBS at a dose of 5.0 mg/kg every two days. The control group was treated with the same volume of PBS instead. Tumor diameters were serially recorded with an electronic caliper every 2 days, and tumor volumes were calculated with the formula: tumor volume (mm^3^) = 0.5×length (mm) ×width^2^ (square mm). Body weights were recorded every 2 days as an indicator of TCS toxicity. Mice were sacrificed on the 12^th^ day of treatment.

### Immunohistochemistry

Immunostaining of tumor tissues was performed as reported elsewhere [7], [35]. Tumor tissues were fixed in 10% formalin overnight and embedded in paraffin. The blocks were cut into 4-micrometer thick slices for different staining. Sections were stained with hematoxylin and eosin (HE) for morphological visualization. On the other hand, sections were incubated with the appropriate primary antibody overnight at dilutions of 1∶50 for anti-cleaved Caspase-3 (Cell Signaling, MA), and 1∶30 for cleaved PARP (sc-23461-R, Santa Cruz Biotechnology, CA), forward by diaminobenzidine/DAB (DAKO Liquid DAB, CA) staining. TUNEL staining was performed using the TUNEL kit (Roche, Germany) as per manufacturer's instructions. All sections were coverslipped and staining was scored by an experienced pathologist blinded to treatment group. The activated caspase-3, cleaved PARP, and TUNEL-positive cells were quantitated as percentage of cells positive from 5 high-powered fields in each section. Slides treated with Isotype-matched IgG but without each primary antibody were served as negative control.

### Statistical analysis

The IC_50_ values were calculated by Sigmaplot software. SPSS 11.0 (SPSS, Chicago) was applied for data analysis. All the data are shown in mean ± standard deviation (S.D.) from at least two independent experiments. For both *in vitro* experiments and *in vivo* mouse experiments, a two-sided Student *t* test was applied for comparison of continuous variables between two groups. Differences were considered significant when the *p* values were<0.05.

## Results

### TCS inhibited cell viability in breast cancer cells

**Figure 1:**
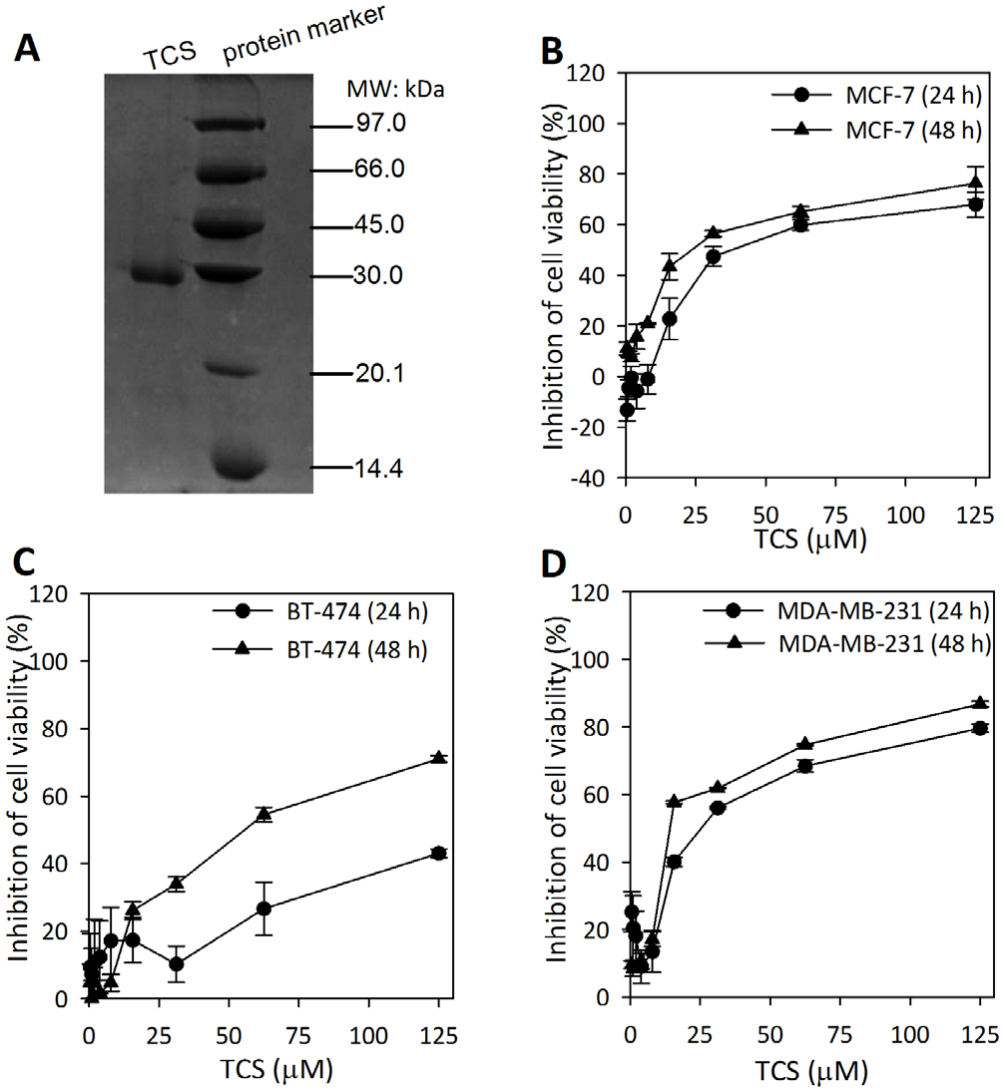
Effect of TCS on the viability of different breast cancer cells. A. The isolated TCS manifested a single band with a molecular mass of 27-kDa. B–D. MCF-7, BT-474, and MDA-MB-231 cells were cultured in the presence of different concentrations of TCS (ranging from 0.49 to 125 µM) for 24 h to 48 h. The remaining cell viability was measured by MTT assay. The results are shown as means ± S.D. of two independent experiments performed in triplicate.

TCS was purified following the method described previously [30]. As shown in Fig. 1A, TCS manifested a single band in SDS-PAGE with a molecular weight of 27 kDa which was in accordance with previous reports [2]. To screen the potential antitumor activity of TCS on breast cancer cells, three representative breast tumor cells were involved, including MCF-7, BT-474, and MDA-MB-231 cell lines. TCS exhibited the ability to time- and dose-dependently inhibit cell viability in all three tested cell lines with different sensitivities (Fig. 1, B to D). For 24 h treatment with TCS, the IC_50_s for the MCF-7, MDA-MB-231and BT-474 tumor cells were 31.6 µM, 20.5 µM, and 130 µM, respectively. After treatment with TCS for 48 h, The IC_50_s for the MCF-7, MDA-MB-231 and BT-474 tumor cells were 25.7 µM, 12.4 µM, and 42.5 µM, respectively. Based on the IC_50_ values, MCF-7 and MDA-MB-231 cells were chosen for further study. Hep G2 cells served as a positive control for the MTT assay as it has been reported to be inhibited by TCS [36]. The IC_50_ of TCS toward Hep G2 cells was 28.6 µM, a value that corroborated previous reports [36].

### TCS caused cell cycle arrest in breast cancer cells

**Figure 2:**
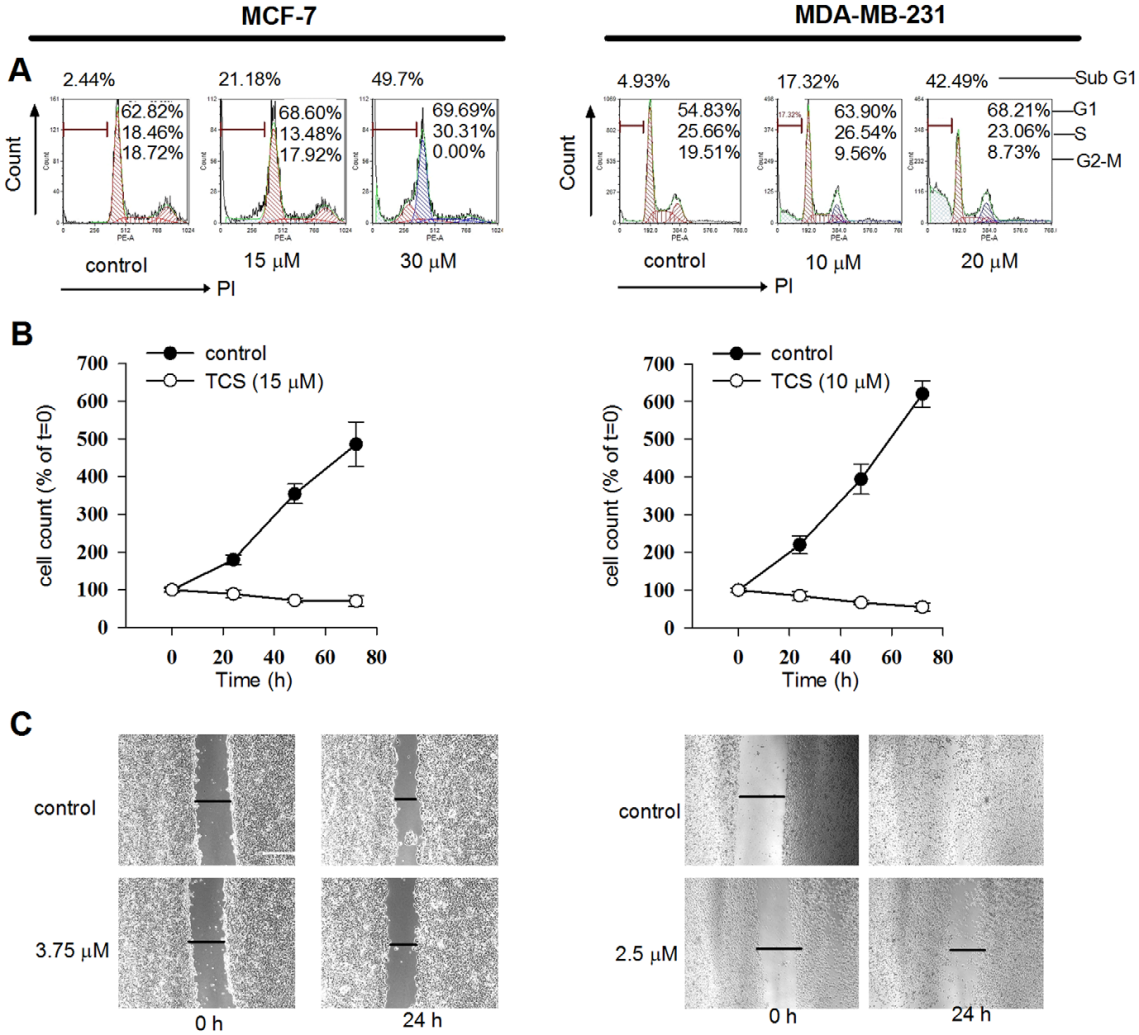
Effects of TCS on cell cycle distribution and proliferation of breast cancer cells. A. TCS induced cell cycle arrest. MCF-7 and MDA-MB-231 cells were treated with different concentrations of TCS (as labeled in the figs.) for 24 h. Cells were harvested, fixed in ethanol, and stained with PI for flow cytometric DNA analysis. Data were analyzed by FCS Express 4 software. B. TCS inhibited cell proliferation. After treatment of MCF-7 and MDA-MB-231 cells for 24 h with 15 µM and 10 µM TCS respectively, cell counting was performed using a haemocytometer following staining with trypan blue solution.

To investigate the potential molecular mechanisms, the effects of TCS on breast cancer cell proliferation were analyzed. Cell cycle analysis showed that 24 h co-culture with TCS induced arrest of proliferation (Fig. 2A). TCS treatment caused cell cycle arrest as shown by changes in the percentage of cells in each sub-phase. On the other hand, there was a significant increase in TCS-induced dead cells, gathered in sub G1 phase, in both MCF-7 (2.44% for control vs. 49.70% for 30 µM TCS-treated group) and MDA-MB-231 cells (4.93% for control vs. 42.49% for 20 µM TCS-treated group). Consistent with this, cell proliferation assay indicated that cells co-cultured with TCS resulted in a significant reduction of the number of growing cells compared with non-treated cells (*p*<0.05, Fig. 2B).

### TCS induced apoptosis in breast tumor cells

**Figure 3:**
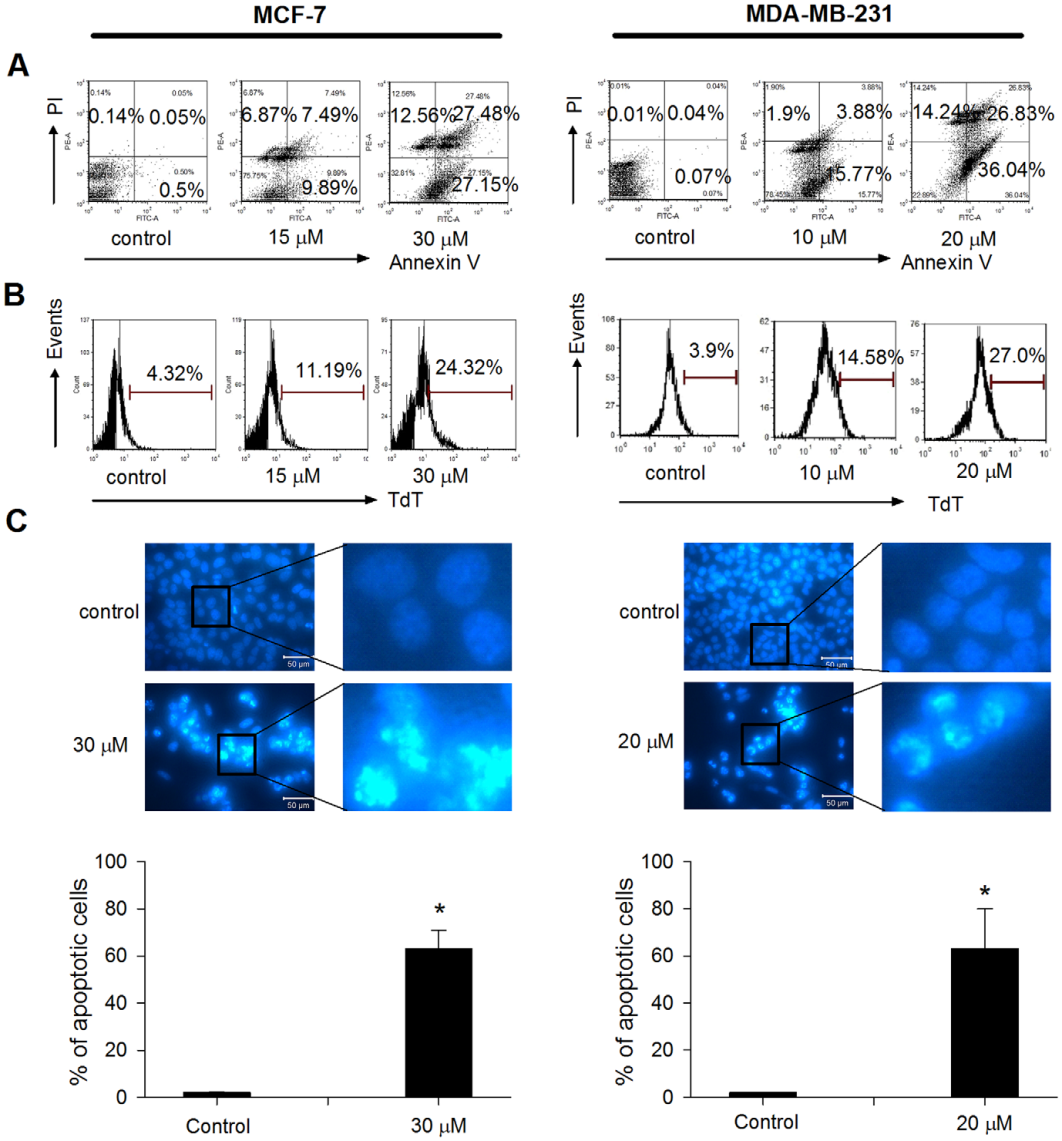
TCS-induced apoptosis in breast cancer cells. A. Cells were treated with TCS at the indicated concentrations for 24 h. Cells were harvested and subsequently stained with Annexin V/PI solution for cell sorting under flow cytometry. Percentages of cells undergoing early apoptosis (right lower quadrant) and late apoptosis (right upper quadrant) were analyzed by FCS Express 4 software. B. Cells were treated with TCS at the indicated concentrations for 24 h, and cells with DNA fragmentation were detected by flow cytometry following TUNEL staining. C. Cells were treated with TCS at the indicated concentrations for 24 h, and nuclear morphological changes were examined by staining with Hoechst 33342 and fluorescence microscopy. Bars, 50 µm. In each panel of Fig. 3C, figures on the right represented fourfold magnification of the rectangular zones in figures on the left.

As the accumulation of cells in sub-G1 phase is indicative of cell death [37], we further assessed whether apoptosis was involved in the phenomenon. As Fig. 3 A indicates, TCS treatment increased the number of apoptotic cells from 0.55% in non-treated group to 55.63% in 30 µM TCS-treated group in MCF-7 cells. Similarly in MDA-MB-231 cells, the number increased from 0.11% in non-treated group to 62.87% in 20 µM TCS-treated group. Furthermore, TUNEL assay was applied to detect DNA fragmentation and the data showed that more cells were undergoing DNA fragmentation after TCS treatment in both MCF-7 cells (4.32% for negative control vs. 24.32% for 30 µM TCS-treated group) and MDA-MB-231 cells (3.9% for negative control vs. 27.0% for 20 µM TCS-treated group) (Fig. 3B). To visualize the nuclear morphological changes in TCS-treated cells, cells were stained with Hoechst 33342 dye. In MCF-7 cells, exposure to 30 µM TCS caused typical morphological changes of apoptosis, such as karyorrhexis, significant nuclear condensation and fragmentation compared with the non-treated group (Fig. 3C, left panel). This phenomenon was also observed in 20 µM TCS-treated MDA-MB-231 cells (Fig. 3C, right panel).

### TCS activated caspase-mediated apoptosis in breast cancer cells

**Figure 4:**
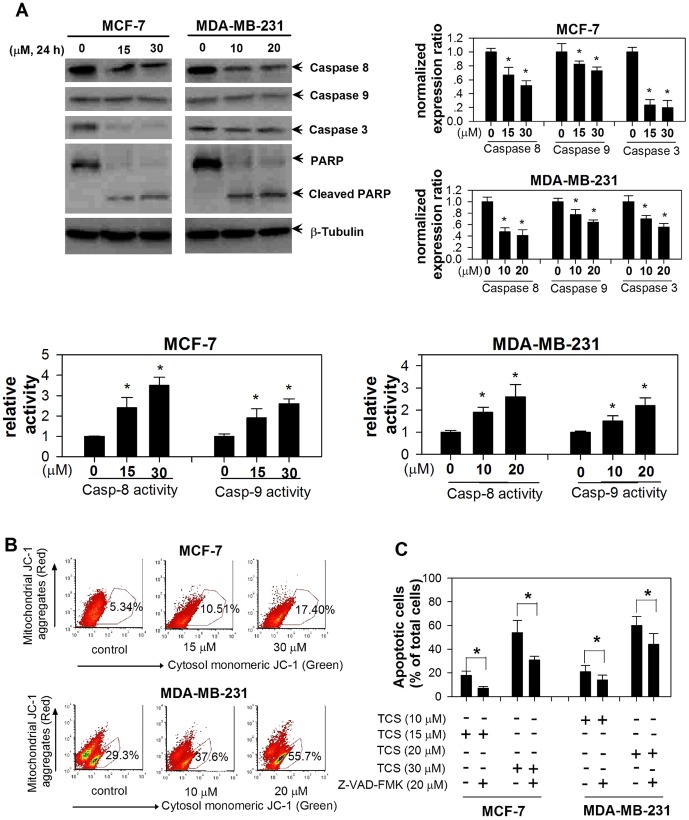
The activation of caspase cascades was involved in TCS-induced cell apoptosis. A. After treatment with TCS at the indicated concentrations, cells were harvested and protein levels of caspase-8, caspase-9, caspase-3, and PARP were determined by western blotting. Right panel, quantitative data from two independent experiments. Lower panel, The caspase-8 and caspase-9 activities were determined by ELISA kits. Asterisk, *p*<0.05 compared with control. B. TCS increased the number of tumor cells undergoing mitochondrial membrane depolarization. Cells were treated with TCS at the indicated concentrations for 24 h, and flow cytometry analysis of the mitochondrial membrane depolarization was carried out after JC-1 staining. C. Z-VAD-FMK pretreatment retarded TCS-induced cell apoptosis. After different treatments as indicated at the bottom of the histogram, cells were harvested, stained with Annexin V-PI, and processed for flow cytometry. Asterisk, *p*<0.05 compared with control.

Apoptosis is always executed in a caspase-8-regulated plasma membrane extrinsic pathway and/or caspase-9-regulated cell damage intrinsic pathway [38]. We then investigated whether one or both the caspase cascades were activated in TCS-induced cell apoptosis. Immunoblot analysis indicated that TCS treatment (15 and 30 µM TCS in MCF-7 cells, 10 and 20 µM TCS in MDA-MB-231 cells) resulted in a dose-dependent activation of the initiator caspases 8 and 9, and the executor caspase 3, and those were followed by proteolytic cleavage of PARP (Fig. 4A). As shown in the right panel of Fig. 4A, the normalized expression levels of remaining caspases 8, 9, and 3 were decreased compared with control (*p*<0.05). Accordingly, there were increased levels of both activated caspase-8 and caspase-9 in TCS-treated group compared with control. It has been proved that apoptosis-inducing agents trigger translocation of caspase 9 from the mitochondria to the nucleus which is a pre-requisite for caspase 9 activation, and apoptosis may be caused by the loss of mitochondrial membrane potential in other mechanisms [39]. Here, JC-1 staining was utilized to detect the damage done to the mitochondrial barrier. In MCF-7 cells, 30 µM TCS increased the cells undergoing mitochondrial membrane damage from a baseline of 5.34% in non-treated group to 17.4% in TCS treated group. In MDA-MB-231 cells, the number expanded from 29.3% for control to 55.7% in 20 µM TCS treated group (Fig. 4B). Additionally, we used a pancaspase inhibitor Z-VAD-FMK (final concentration 20 µM) to pre-treat both breast cancer cells for 1 h before adding TCS. Compared with TCS group, Z-VAD-FMK pre-treatment decreased the percentage of apoptotic cells (*p*<0.05 in both MCF-7 and MDA-MB-231 cells, Fig. 4C).

### Efficacy of TCS to inhibit tumor growth in nude mice

**Figure 5:**
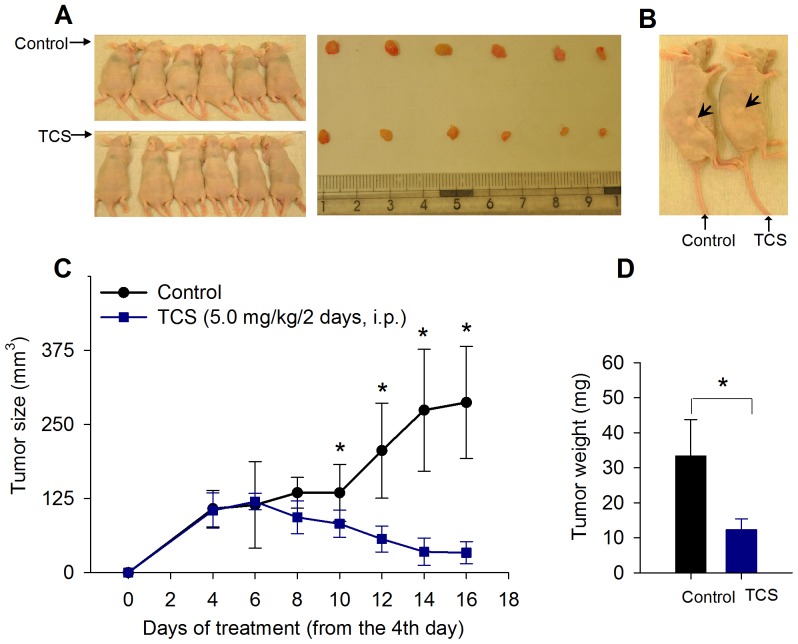
TCS inhibits MDA-MB-231 xenograft growth in nude mice. A. Twelve female BALB/c nude mice received an injection of MDA-MB-231 cells and were divided into 2 groups. TCS was administered at a dose of 5.0 mg/kg every other day for a total of 7 injections. On day 16, mice were sacrificed and excised tumors are shown in the right panel. The dose applied had no detectable toxicological effects on nude mice, such as changes in body weight. B. A representative comparison between a TCS-treated mouse and a PBS-treated control mouse. C. TCS significantly reduced tumor volume from the 6^th^ day of treatment onward. D. The mean tumor weight in TCS group was smaller than that of control. For asterisks in C and D, *p*<0.05.

After revealing the antitumor potential of TCS in breast cancer cells *in vitro*, we went on to determine whether the effect was also observed *in vivo*. After subcutaneous inoculation with MDA-MB-231 cells into the right flanks of nude mice for 4 days, tumor xenografts were detectable and the mice were assigned to two groups with 6 in each group: control treated with PBS, and TCS-treated group (5.0 mg/kg body weight TCS, i.p. every two days). The TCS dose applied had no detectable toxicity in both groups since there were no statistically significant effects on body weight (control vs. TCS group, 23.5±1.4 g vs. 22.7±2.6, *p*<0.05), behavior, and appearance (Fig. 5A, left panel). On the sixth day of TCS treatment (day 10 post-tumor injection), the treatment brought about a significant reduction in the tumor volume compared with the control. The tendency was persistent and more significant on the 12^th^ day of TCS treatment (day 16 post-tumor injection) (Fig. 5C). On day 16, mice were sacrificed and the tumors were resected. Compared with control, TCS significantly decreased the mean tumor weight (12.27±3.13 vs. 33.39±10.32, *p*<0.05) (Fig. 5A left panel, and Fig. 5D). A representative comparison is shown in Fig. 5B.

### Immunohistochemistry

**Figure 6:**
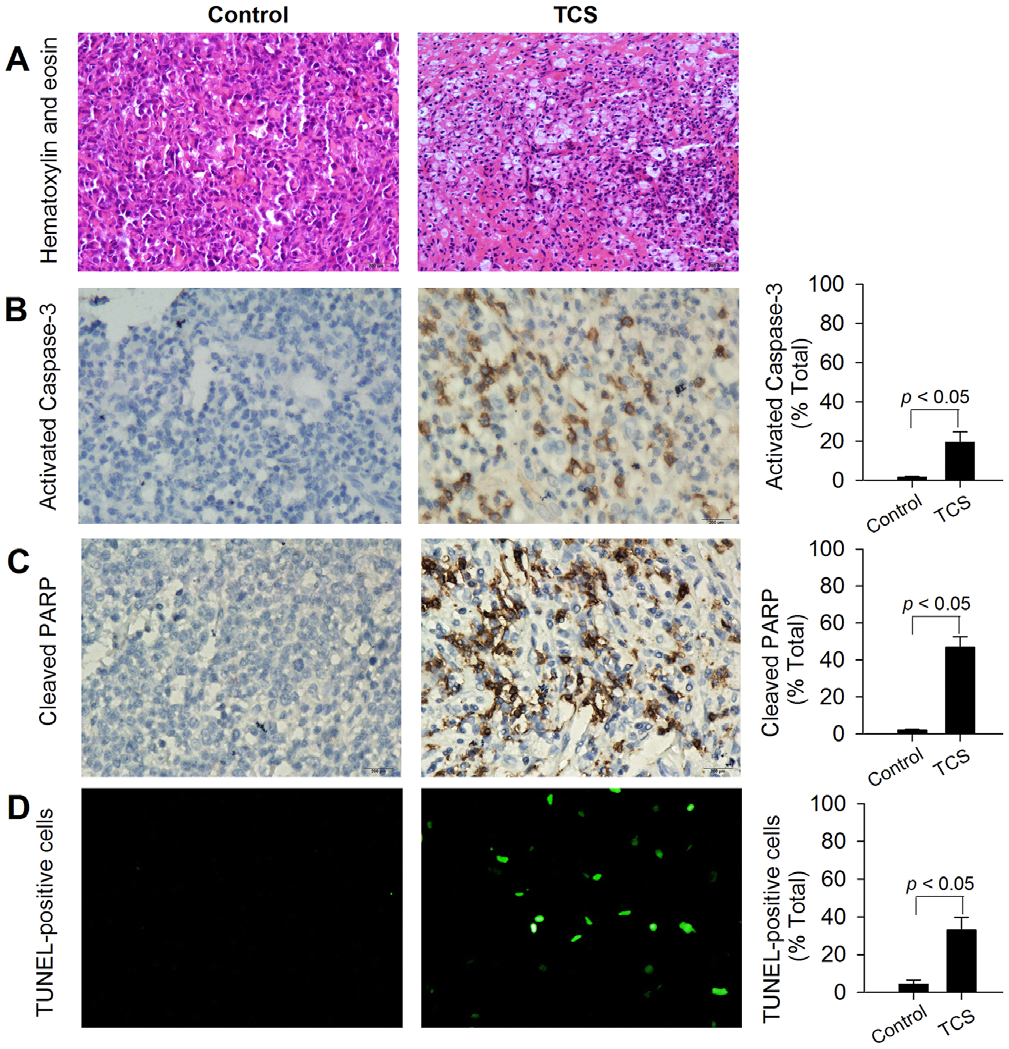
TCS-induced necrosis and apoptosis in MDA-MB-231 xenograft. MDA-MB-231-bearing nude mice were treated with a TCS dose of 5.0 mg/kg every other day by i.p. injection for a total of 7 injections. On day 16 post-cell injection, tumors were excised carefully, and immunohistochemistry studies were performed as detailed in Materials and methods. Positive cells in each study were scored semi-quantitatively. A. HE staining. B. Tumor slides were examined for apoptosis by activated caspase-3. Representative images from both groups are shown, and positive cells were stained brown. C. Tumor slides were examined for apoptosis by cleaved PARP. Representative images from both groups are shown, and positive cells were stained dark brown. D. TUNEL assay was used for detecting DNA fragmentation. Representative images from both groups are shown with TUNEL-positive cells colored green. Microscopic magnification 200×.

Immunohistochemistry analysis of tumor tissues further corroborated that necrosis and apoptosis had occurred in TCS-treated tumors (Fig. 6). In comparison with control, HE staining showed that TCS induced nuclear shrinkage, necrosis, and inflammatory cell infiltration in treatment group (Fig. 6A). We have also observed significantly increased activated caspase-3 and the downstream PARP cleavage in TCS-treated group, whereas these stained cells were scare in the control group (Fig. 6B and 6C). Moreover, as Fig. 6D indicates, TCS treatment resulted in over 7-fold increase of TUNEL-positive cells as compared with control (4.5±2.1 vs. 33.0±6.8, *p*<0.05).

## Discussion

Breast cancer is a leading gynecological disease, and though its mortality has been significantly lessened due to adjuvant therapies, the increased incidence, high relapse as well as metastasis after treatment make breast cancer still a major clinical hurdle [27]. Besides surgical resection, current treatments for breast cancer comprise radiotherapy, hormonal and biological therapies, and chemotherapy, for the latter the investigations of synthesized and natural medicinal components are actively processed [31], [40], [41]. In recent years, new components or common drugs in new combinations targeted on breast tumor cells have been reported [31], [42]. In this study, we showed that TCS exhibited potent antitumor activity toward breast cancers in both cell culture and mouse models.

Three breast cancer cell lines, including two estrogen-dependent breast cancer cell lines are MCF-7 cells and highly metastatic BT-474 cells, and estrogen-independent MDA-MB-231 cells, were employed in this study. TCS inhibited cell viability in all three tested cell lines in the following ranking of potency:MDA-MB-231>MCF-7>BT-474 cells. MDA-MB-231 and MCF-7 cells were chosen for further studies. It was revealed that TCS exhibited both cytostatic (Fig. 2B) and cell-death inducing (Fig. 2A, sub G1 numbers) activities which in turn contributed to the inhibition of cell viability in both types of breast cancer cells. This cytostatic activity was at least partially contributed by cell cycle arrest. It seems that TCS may induce G1 phase arrest in breast cancer cells as it did in human lung cancer A549 cells [19]. Further investigations on the effect of TCS on some G1 cell cycle-regulating proteins, such as cyclin D1 and phosphor-Rb [43], are warranted. Furthermore, flow cytometric analysis using Annexin V/PI showed that TCS could dose-dependently induce both early apoptosis and late apoptosis in MDA-MB-231 and MCF-7 cells. In accordance with this, stereotypical apoptotic features were noticed, including karyorrhexis, chromatin condensation and internucleosomal DNA fragmentation (Fig. 3 B–C). The results were reminiscent of the action of other natural chemotherapeutic components toward breast cancer cells. For instance, the ribonuclease from *Momordica charantia* seeds (RNase MC-2) manifested the same effects on MCF-7 cells [31].

Caspases are the principal effectors of apoptosis involved in pathways such as caspase-8-regulated extrinsic and caspase-9-regulated intrinsic pathways. The caspase-9 pathway links mitochondrial damage to caspase activation, and serves as an index of damage in mitochondrial membrane function [39]. Furthermore, the co-downstream member caspase-3 is an executor of DNA fragmentation and apoptosis as exemplified by cleavage of PARP during cell death [44]. Our results showed that TCS dose-dependently activated caspase-8 and caspase-9, each at the apex of its pathway, following which came caspase-3 activation and cleavage of PARP (Fig. 4A). As expected, TCS-induced mitochondrial membrane depolarization was observed. Moreover, pre-treatment of tumor cells with the cell-permeating pancaspase inhibitor Z-VAD-FMK partially rescued TCS-induced apoptosis which corroborates the involvement of caspase cascades in TCS-induced apoptosis. The action of TCS toward tumor cells coincides with another RIP, *M. charantia* lectin (MCL). This type-II RIP has been reported to induce apoptosis by activation of both caspase-8 and caspase-9 regulated pathways in nasopharyngeal carcinoma cells [6], [7].

The antitumor efficacy of TCS in *in vivo* systems was substantiated by results from MDA-MB-231 bearing nude mice. Every other day, TCS was administered intraperitoneally at a dose of 5.0 mg/kg body weight. Compared with control, TCS treatment abated both tumor volume as well as tumor weight from the 6th day of treatment. This *in vivo* antitumor effect correlated with increased levels of apoptosis in the tumors of TCS-treated group, as supported by the increase of activated caspase-3, cleaved PARP, and DNA fragmentation (TUNEL-positive cells). Interestingly, necrosis was also detected in TCS-treated group compared to the control group. These data suggest that reduction of tumor proliferation by induction of apoptosis is a mechanism of the *in vivo* antitumor activity of TCS toward MDA-MB-231 xenograft. More importantly, previous clinical studies on the application of TCS against other human diseases showed that it was also effective, and the effective dose level was safe and relatively well tolerated, though with some acceptable side effects [2], [4], [9], [10], [11]. Protein engineering studies on TCS, such as PEGylation and the construction of immunotoxin, may help to increase its drug specificity as well as reduce its side effects [45], [46].

In particular, several natural RIPs have been the subject of studies that have documented potential medicinal applications of RIPs. The 30-kDa MAP30 is a type I RIP isolated from *M. charantia* which manifestsvsequence homology with TCS to some extent [2], [6]. Lee-Huang and coworkers reported that MAP30 inhibited cell proliferation and the expression of HER2 in MDA-MB-231 cells. More importantly, treatment of MDA-MB-231 bearing SCID mice with TCS at a dose of 10 µg/injection EOD for a total of ten injections, resulted in pronounced prolongation of survival, and nearly one quarter of the mice remained tumor-free for 96 days [47]. Furthermore, the abovementioned MCL was also active *in vivo* in a CNE-2 xenograft tumor model. It inhibited tumor growth by inducing apoptosis of tumor cells [7]. TCS manifested cytotoxicity in both MDA-MB-231 cells and MCF-7 cells. Our next step is to test the antitumor effect of TCS on MCF-bearing nude mice to further extend the potential medicinal application to estrogen-dependent breast cancer patients.

An interesting question is whether the apoptosis-inducing activity of TCS is attributed to its ribosome inactivating activity. Based on the information provided by previous studies on other RIPs, it seems that the apoptosis-inducing activity of a RIP could be contributed by ribosomal damage, but not restricted by its ribosome inactivating activity. When investigating the apoptosis-inducing activity of the well-known ricin on U937 cells, Komatsu and colleagues found that ribosome inactivation may be responsible for the apoptotic events induced by ricin, since the application of different protein synthesis inhibitors did not significantly alleviate the effects [48]. On the other hand, an attractive study done by Lee-Huang and coworkers showed that proteolytic fragments of MAP30 retained competitive antitumor activity as the intact native form. However, these bioactive fragments were devoid of ribosome inactivating activity [49]. Interestingly, TCS shares 59% sequence similarity with MAP30 [2]. MAP30 has also shown antitumor activity similar to that of TCS elucidated here. The antitumor activity of MAP30 on Hep G2 cells is attributed to activation of both extrinsic caspase-8 and intrinsic caspase-9 pathways, and phosphorylation of both Akt and some MAPK members [50].

It is probable that an RIP may trigger multiple death signaling pathways, and our current results clearly indicate that both extrinsic and intrinsic caspase cascades were involved in TCS-induced apoptosis in breast cancer cells. Whether inactivation of ribosomes was another cause of cell death needs further investigation. Furthermore, the specificity of TCS on tumor cells and its cellular entry mechanism have also been studied previously. The way for TCS to enter into the tumor cells is associated with the binding on two receptor proteins (with a molecular weight of 50 and 60 kDa, respectively), and/or depending on a2-macroglobulin receptor/low density lipoprotein receptor related protein (LRP)-mediated endocytosis [2].

In this study, TCS was administered intraperitoneally at a dose of 5.0 mg/kg body weight. The dose applied is acceptable since the mice did not lose weight during the course of treatment. Recently, a large clinical study indicated that TCS was effective against ectopic pregnancy in 140 women (28.9 years, range 18 to 44) at the dose of 1.8 mg/person using intramuscular injection. There were no significant side effects for most of the patients and it seems to have no effects on their subsequent pregnancy as well as giving birth to a healthy baby [51]. However, the efficiency as well as recommended dosage of TCS on breast cancer patients will depend on further clinical trials. Recently, Dakeng and coworkers showed that cucurbitacin B (a triterpene extracted from the closely related species, *Trichosanthes cucumerina* Linn) displayed anticancer effects in breast cancer cells [52]. The antitumor activity of TCS shown here could not be attributed to the possible presence of contamination with cucurbitacin B and other small compounds in the purified TCS, since dialysis tubings (with 3.5–5 kDa molecular weight cut-off, from Biotech Cellulose Ester Membrane) were used for extensive dialysis of the purified protein against a copious amount of double distilled water (>500 times the volume of protein sample with three changes of fresh water over 24 hours). In conclusion, TCS manifested the ability to induce apoptosis in both estrogen-dependent MCF-7 cells as well as estregen-independent MDA-MB-231 cells in *in vitro* and/or *in vivo* experiments. Proteolytic processing of initiator caspases as well as executor caspase, and subsequent spawned apoptosis contributed to TCS-induced apoptosis. Besides the function as an abortifacient, and application in the treatment of hydatidiform moles and invasive moles, TCS may provide a plethora of treatments or even for the development of a novel therapy combined with other chemotherapeutic measures for both estrogen-dependent and estrogen-independent breast cancers. Further clinical trials are warranted.
